# Low skeletal muscle radiodensity is the best predictor for short-term major surgical complications in gastrointestinal surgical cancer: A cohort study

**DOI:** 10.1371/journal.pone.0247322

**Published:** 2021-02-19

**Authors:** Ana Lúcia Miranda de Carvalho, Maria Cristina Gonzalez, Iasmin Matias de Sousa, Isabel Pinto Amorim das Virgens, Galtieri Otavio Cunha de Medeiros, Marília Nelo Oliveira, Jeane Cristina Alves de Souza Dantas, Ana Paula Trussardi Fayh

**Affiliations:** 1 Postgraduate Program in Nutrition, Health Sciences Center, Federal University of Rio Grande do Norte, Natal, State of Rio Grande do Norte, Brazil; 2 Postgraduate Program in Health and Behavior, Catholic University of Pelotas, Pelotas, State of Rio Grande do Sul, Brazil; 3 Postgraduate Program in Health Sciences Center, Federal University of Rio Grande do Norte, Natal, State of Rio Grande do Norte, Brazil; 4 Nutrition Department, Luiz Antônio Hospital, Liga Contra o Câncer, Natal, State of Rio Grande do Norte, Brazil; Humanitas Clinical and Research Center - IRRCS, ITALY

## Abstract

The aim of this study was to evaluate whether body composition, muscle function, and their association are predictive factors for short-term postoperative complications in patients with gastric and colorectal cancer. A prospective cohort study was conducted with patients undergoing resection of gastric and colorectal tumors. Nutritional status was assessed using Patient-Generated Subjective Global Assessment (PG-SGA) and anthropometric techniques. Low handgrip strength (HGS) was observed when <16kg for women, and <27kg for men. Computed tomography images were used to measure visceral adipose tissue, skeletal muscle index (SMI), and skeletal muscle radiodensity (SMD). Complications of grade II or above (according to Clavien-Dindo’s classification) were considered in a follow-up period of up to 30 days after surgery. Major complications were defined when they reached grade III or above. A total of 84 patients were analyzed (57.1% female, 59.7 ± 12.6 years) and 19% were diagnosed with low HGS + low SMI or SMD. Postoperative complications occurred in 51.2%, and these patients presented significantly longer duration of surgery and hospital stay. Major complications were observed in 16.7% of the total number of patients. Binary logistic regression adjusted by age, sex, and tumor staging showed that low SMD, low HGS + low SMI or SMD, and obesity were independent risk factors for postoperative complications, but only low SMD was an independent risk factor for major postoperative complications. Low SMD is an independent risk factor for short-term major complications following surgery in patients with gastric and colorectal cancer.

## Introduction

Cancer is one of the leading causes of death worldwide, with an upward incidence due to population growth and aging, as well as the adoption of already proven carcinogenic habits such as smoking, inadequate diet, and physical inactivity [1, 2]. The global estimates by the International Agency for Research on Cancer (IARC) show that more than 18 million new cases of cancer were diagnosed in 2018, and about 9 million deaths occurred [3]. In Brazil, 625 thousand new cases are predicted to occur in each year in the 2020–2022 period, with gastric and colorectal tumors among the top 10 most occurring types [4].

Surgery is considered the cornerstone in the treatment of gastric and colorectal cancer, allowing staging of the disease, verifying its extension, and removing all visible tumors. However, major surgeries are associated with a higher frequency of postoperative complications and greater morbidity, with a negative impact on short and long-term outcomes [5]. To prevent or minimize the occurrence of such complications, the impact of nutritional status, body composition, and functional capacity alterations in the pre-surgical period has been investigated [6–8].

Malnutrition is frequent in patients with gastric and colorectal tumors [9–11] due to the combination of effects related to disease progression, host response to the tumor(s), treatment symptoms and the direct effect of mechanical obstruction caused by the tumor, with consequent malabsorption of nutrients [12, 13]. Muscle wasting associated with malnutrition are frequent in cancer patients and may lead to the development of secondary sarcopenia, associated with adverse outcomes [14–16]. Sarcopenia can occur even in the absence of weight and fat loss, and it can, therefore, go undetected in patients who are overweight or obese [17–19]. Also, sarcopenia is associated with negative outcomes, mainly a higher rate of post-surgical complications, longer hospital stays and a worse prognosis after cancer surgery [6, 15].

Various techniques may be used to estimate body composition, but analysis of Computed Tomography (CT) images obtained as part of the routine treatment has emerged as the preferred one [17]. CT images can evaluate skeletal muscle mass and the amount and distribution of adipose tissue (subcutaneous vs. visceral) and tissue-specific radiodensity values [8, 20, 21]. Reduced skeletal muscle radiodensity (SMD), referred to as myosteatosis, reflects intramuscular fat infiltration, and it can also directly affect survival [22, 23] and prognosis of cancer patients [24, 25].

Although the role of muscle mass in the prognosis of cancer patients undergoing surgical treatment is well established in the literature [8, 15, 26, 27], a discrepancy between studies in determining the degree of severity of the complications and of the muscle mass impairment contributes to a confusing interpretation of the results. Furthermore, recent systematic reviews with meta-analyses showed that the effect of low muscle mass on the risk of complications and mortality in cancer patients may vary according to the type of cancer or complication severity [28, 29], but both studies used only skeletal muscle index (SMI) to verify the effect, disregarding other CT-determined body components, as SMD and visceral adipose tissue. Our hypothesis is that, as in primary sarcopenia, the presence of an impaired physical function, such as low handgrip strength (HGS), combined with other CT-determined muscle abnormalities (low SMD or low SMI) may represent more severe sarcopenia and improve the risk prediction, making it more sensitive to identify the higher-risk patient prone to short-term and more severe complications. Thus, the aim of this study was to evaluate whether body composition, muscle function, and CT-determined muscle abnormalities are predictive factors for postoperative complications in patients with gastric and colorectal cancer.

## Materials and methods

### Design and subjects

A prospective cohort study of patients undergoing elective open gastric and colorectal cancer resection was conducted between December 2017 and December 2018 in a single center, in Brazil. Patients with histopathological gastric and colorectal cancer diagnosis were included. Patients without CT scans available for, at least, three months before the date of the surgical procedure or whose analysis was impaired (absence of the third lumbar vertebra in the image, presence of ascites or edema) or undergoing palliative surgery (only exploratory laparotomy and biopsy) were excluded. The sample size was calculated according to a previous study that found 27.4% of total postsurgical complications in 376 colorectal cancer patients [8]. Considering a standard error of 10%, it was necessary to evaluate, at least, 76 patients (G*Power^®^, version 3.1.9.2; Institute for Experimental Psychology in Dusseldorf, Germany). The study protocol was approved by the Research Ethics Committee from the Onofre Lopes University Hospital (protocol number 73316117.8.0000.5292) and all participants signed the written informed consent form in the admission before surgery.

### Procedures

Clinical and demographic data were obtained from the digital records at the Hospital one day before the surgical procedure: age, sex, ethnicity, presence of comorbidities (diabetes and/or hypertension), primary site of the tumor, neoadjuvant treatment with chemotherapy and/or radiotherapy and functional capacity by ECOG-PS (Eastern Cooperative Oncology Group Performance Status). Information about the duration and type of surgery performed, length of hospital stay and the occurrence of post-surgical complications was collected from medical records at the end of the follow-up. Patients were staged according to the eighth edition of the American Joint Committee on Cancer (AJCC) staging manual [30], based on the histopathological report.

### Nutritional status and muscle strength

Nutritional status was assessed using the anthropometric technique and subjective evaluation. Body Mass Index (BMI) was calculated from weight and height squared in meters, and then, patients were classified according to the WHO criteria [31]. An inelastic tape (Sanny^®^, Brazil) was used for calf circumference (CC) measurement, and individuals were seated with their legs positioned at a 90° angle. The cut-off point validated for this population was adopted, which indicates a low CC when the value is equal or less than 33 cm for women and 34 cm for men [32].

The Patient-Generated Subjective Global Assessment (PG-SGA) was also applied, in which the patient is classified as well-nourished (PG-SGA A), suspected or moderately undernourished (PG-SGA B), or severely malnourished (PG-SGA C) [33, 34]. Patients classified as B and C in the present study were grouped and classified as malnourished.

Cachexia was defined according to the criteria proposed by Fearon [35] of involuntary weight loss > 5% over the last 6 months (in the absence of simple starvation), or BMI less than 20kg/m^2^ and any weight loss > 2% in the last 6 months, or low SMI associated with any weight loss > 2%.

Handgrip strength (HGS, kg) was measured one day before surgery, using a calibrated dynamometer (Jamar^®^) with the dominant hand. Patients were instructed to sit on a bed holding the dynamometer in their hand comfortably, with their arm resting at a 90° angle with the forearm and were then instructed to squeeze the dynamometer handle at maximum strength for at least 3 seconds. After three attempts with a minimum rest period of 60 seconds between them, the highest recorded value was used as maximum muscular strength [36]. The categorization of low muscle strength (an indicator of muscle function) was performed according to the following cut-off points: < 16kg for women and < 27kg for men [37].

### Computed tomography images

CT images available in the hospital system (up to 90 days prior to the surgical procedure) were used for the analysis of body composition. A single transverse slice CT image at the third lumbar vertebra (L3) was analyzed using the Slice-O-matic software (v5.0, Tomovision^®^, Montreal, Canada). The tissues were demarcated using the Hounsfield Units (HU) thresholds of -29 to +150 for skeletal muscle and -150 to -50 for Visceral Adipose Tissue (VAT). The Skeletal Muscle Index (SMI, cm^2^/m^2^) was calculated through the total muscle area, corrected by the body surface. Skeletal muscle radiodensity quantifies the average radiation attenuation rate (HU) and it is a radiological measure of the extent of lipid contained within the muscle. Low SMI and low SMD (quantitative and qualitative muscle abnormalities) were defined according to the cut-off points proposed by Martin et al. [25], in a cohort study with adult patients with a diagnosis of gastrointestinal or lung cancer: for men, SMI < 43 cm^2^/m^2^ when BMI < 25 kg/m^2^ or SMI < 53 cm^2^/m^2^ when BMI ≥ 25 kg/m^2^; for women, SMI < 41 cm^2^/m^2^, regardless of BMI. Low SMD < 41 HU when BMI < 24.9 kg/m^2^ and < 33 HU when BMI ≥ 25 kg/m^2^ for both genders. These cutoff points were chosen for being the most widely used in the literature and based on the similarities between patients (gastrointestinal cancer with advanced staging). Visceral obesity was evaluated from the amount of VAT at the L3 level and defined from the cut-off point of 163.8cm^2^ for men and 80.1cm^2^ for women, proposed by Doyle et al. [38] in a population of patients with gastrointestinal cancer, similar to the present study. All analyzes were performed by a single trained expert, blinded to the outcome.

### Outcome

The postoperative course was observed for 30 days after surgery. In case of discharged, these monitoring was carried out by observing whether there was re-hospitalization within this period for treatment of surgery complications, based on the records in the electronic hospital record. The Clavien-Dindo Classification (CDC) [39] was used, which classifies the surgical complications in degrees from I to V according to the severity. The translated and adapted version of the scale to Brazilian Portuguese was used in our study [40]. Complications of grade II or above were considered in this study, and they include infectious processes treated with antibiotics, need for blood transfusion and parenteral nutrition. Complications of grade III or higher were considered severe, including surgical re-interventions for correcting fistulas, intra-abdominal abscess and evisceration, admissions in ICU for treating abdominal sepsis, in addition to death.

### Statistical analysis

Normal distribution of the continuous variables was verified by the Kolmogorov-Smirnov test. The groups with and without postoperative complications were compared using the statistical package SPSS version 25.0 (IBM^®^, Chicago, IL, USA). Continuous data with a normal distribution were compared using Student’s t-test for independent samples and are expressed as mean and standard deviation (SD). Data with non-normal distribution are expressed as median or interquartile range (IQR) and were tested for statistical differences by the Mann-Whitney U test. The categorical data are expressed in absolute and relative frequency (%). Pearson’s chi-squared test was used for the bivariate association analysis between postoperative complications and risk factors, and relative risk was calculated with a 95% confidence interval. The body composition and nutritional status variables that presented p < 0.20 were tested in a logistic binary regression, adjusted for confounding variables (age, sex and tumor stage) to evaluate the Odds Ratio for post-surgical complications. A p-value < 0.05 was considered significant.

## Results

The overall characteristics of the sample are shown in Table 1. Eighty-four (84) patients with mean age of 59.7 ± 12.58 years were included in the study, the majority being female, and non-Caucasian. Of the total, 65.5% had colorectal cancer, and stages II and III were the most prevalent. Only 17.8% had ECOG-PS higher than 1 and 23.8% of the patients had undergone neoadjuvant chemotherapy and radiotherapy. The median length of hospital stay was of 5 days (IQR: 4.0–8.7), and the time between CT and the surgery was of 34 days (IQR: 23.2–48.7). More than a half of the patients (51.2%) had complications of grade II or higher during the follow-up period and 14 patients (16.7%) had severe complications (grade III or higher), and 6 patients (7.2%) died. No statistically significant differences were found when comparing the prevalence of low SMD between patients with or without previous treatment (15% vs 17,2% respectively, p = 0.053) and tumor site (17.2% for gastric cancer vs 16.4% for colorectal cancer, p = 0.011). There was also no statistically significant difference between low SMI between patients with or without previous treatment (10% vs 20,3% respectively, p = 1.105) and tumor site (13,8% for gastric cancer vs 20% for colorectal cancer, p = 0.499).

**Table 1:** Demographic and clinical characteristics of patients who underwent gastric and colorectal cancer surgery (n = 84).

Characteristics	n	%
**Sex**		
**Female**	48	57.1
**Male**	36	42.9
**Ethnicity**		
**Caucasian**	62	73.8
**Non-Caucasian**	22	26.2
**Tumor site**		
**Gastric**	29	34.5
**Colon/rectum**	55	65.5
**TNM stage**		
**I**	18	21.4
**II**	21	25.0
**III**	26	31.0
**IV**	16	19.0
**Unknown**	3	3.6
**ECOG-PS**		
**0**	38	45.2
**1**	31	36.9
**2**	9	10.7
**3**	6	7.1
**Neoadjuvant treatment**		
**Yes**	20	23.8
**No**	64	76.2
**Duration of surgery (min)**		
**<120**	11	13.1
**120–239**	50	59.5
**≥240**	23	27.4
**Postoperative complications ≥ grade II**		
**Yes**	43	51.2
**No**	41	48.8
**Postoperative complications ≥ grade III**		
**Yes**	14	16.7
**No**	70	83.3

Data expressed in absolute and relative frequency (%).

ECOG-PS: Eastern Cooperative Oncology Group Performance Status.

Bivariate analysis (Tables 2 and 3) showed that postoperative complications were associated with the site of the tumor being in the stomach (p = 0.018), tumors of stage III and IV (p = 0.033), presence of obesity (p = 0.011), low SMD (p = 0.025), low function (HGS) + muscle impairment (low SMI or SMD) (p = 0.034), and visceral obesity (p = 0.030). No significant association was observed between postoperative complications and the other variables. In addition, patients who developed postoperative complications presented higher amounts of VAT (123.7 ± 84.5 cm^2^ *vs* 89.7 ±64.6 cm^2^, p = 0.042), longer duration of surgery (median of 195 min *vs* 150min, p = 0.002) and length of hospital stay (median of 8 days *vs* 5 days, p <0.001).

**Table 2:** Demographic and clinical characteristics of patients who underwent gastric and colorectal cancer surgery and associations with 30-day postoperative complications (n = 84).

Characteristics	n (%)	Complications n (%)	RR (95% CI)	p
**Sex**				0.801
Male	36 (42.9)	19 (52.8)	1	
Female	48 (57.1)	24 (50.0)	0.95 (0.62;1.44)	
**Age**				0.996
< 60 years	41 (48.8)	21 (51.2)	1	
> 60 years	43 (51.2)	22 (51.2)	1.00 (0.66;1.52)	
**Comorbidities**				0.666
None	41 (48.8)	20 (48.8)	1	
Diabetes/hypertension	43 (51.2)	23 (53.5)	1.10 (0.72;1.67)	
**TNM stage**^a^				**0.033**
I-II	39 (46.4)	16 (37.8)	1	
III-IV	42 (50.0)	25 (61.9)	1.64 (1.02;2.63)	
**Neoadjuvant treatment**				0.903
No	64 (76.2)	33 (51.6)	1	
Yes	20 (23.8)	10 (50.0)	0.97 (0.59;1.60)	

RR: relative risk; 95% CI: 95% confidence interval.

^a^TNM stage was unknown in three patients.

**Table 3:** Pre-operative nutritional and body composition parameters of patients who underwent gastric and colorectal cancer surgery and associations with 30-day postoperative complications (n = 84).

Characteristics	n (%)	Complications n (%)	RR (95% CI)	P
**PG-SGA**				0.346
B or C	33 (39.3)	19 (57.6)	1	
A	51 (60.7)	24 (47.1)	0.82 (0.54;1.24)	
**Calf circumference**				0.503
Normal	70 (83.3)	35 (81.4)	1	
Low^a^	14 (16.7)	8 (18.6)	1.14 (0.69;1.90)	
**Low HGS**^b^				0.204
No	58 (69.0)	27 (46.6)	1	
Yes	26 (31.0)	16 (61.5)	1.32 (0.88;1.99)	
**Cachexia**				0.467
No	52 (61.9)	25 (48.1)	1	
Yes	32 (38.1)	18 (56.3)	1.17 (0.77;1.77)	
**Low SMI**^c^				0.186
No	69 (82.1)	32 (45.7)	1	
Yes	15 (17.9)	10 (66.7)	1.39 (0.90;2.15)	
**Low SMD**^d^				**0.025**
No	70 (83.3)	33 (47.8)	1	
Yes	14 (16.7)	11 (78.6)	1.72 (1.18;2.50)	
**Low Function + Muscle impairment**^e^				**0.034**
No	68 (81.0)	31 (45.6)	1	
Yes	16 (19.0)	12 (75.0)	1.65 (1.12;2.42)	
**Obesity**^f^				**0.011**
No	66 (78.6)	29 (43.9)	1	
Yes	18 (21.4)	14 (77.8)	1.77 (1.23;2.56)	
**Visceral obesity**^g^				**0.030**
No	47 (56.0)	19 (40.4)	1	
Yes	37 (44.0)	24 (64.9)	1.70 (1.03;2.80)	

PG-SGA: Patient-Generated Subjective Global Assessment; HGS: handgrip strength; SMI: skeletal muscle index; SMD: skeletal muscle radiodensity; RR: relative risk; 95% CI: 95% confidence interval.

^a^Low calf circumference: ≤ 33 cm for females and ≤ 34cm for males;

^b^Low HGS: < 16kg for females and < 27kg for males;

^c^Low SMI: < 43 cm^2^/m^2^ (BMI < 25 kg/m^2^) or SMI < 53 cm^2^/m^2^ (BMI ≥ 25 kg/m^2^) for males; SMI < 41 cm^2^/m^2^ for females;

^d^Low SMD: SMD < 41 HU (BMI < 24.9 kg/m^2^) or < 33 HU (BMI ≥25 kg/m^2^);

^e^Low function + muscle impairment: low HGS + low SMI or low SMD;

^f^Obesity: BMI ≥ 30 kg/m^2^;

^g^Visceral obesity: VAT > 80.1cm^2^ for females and > 163.8 cm^2^ for males.

Logistic binary regression was used to analyze the association between body composition variables and complications within 30 days after surgery, adjusted or not for confounding factors (Tables 3 and 4). For complications of grade II or above (Table 4), individuals with obesity (BMI ≥ 30 kg/m^2^), visceral obesity, low SMD and low function + muscle impairment presented more chances of having postoperative complications, even with the adjustment for confounding variables (age, sex, and tumor staging).

**Table 4:** Logistic binary regression model analysis of factors associated with postoperative complications within 30 days after surgery in patients with gastric and colorectal cancer (n = 84).

	Complications Grade ≥ 2 (n = 43; 51.2%)	
Independent variables	Unadjusted	Adjusted^a^
	OR (95% CI)	OR (95% CI)
**BMI**	**p = 0.016**	**p = 0.014**
BMI < 30 kg/m^2^(n = 18)	1	1
BMI ≥ 30 kg/m^2^ (n = 66)	4.47 (1.33;15.02)	5.16 (1.39;19.21)
**Visceral obesity**	**p = 0.026**	**p = 0.020**
VAT ≤ 163.8/80.1 cm^2^ (n = 56)	1	1
VAT > 163.8/80.1cm^2^ (n = 37)	1.70 (1.03; 2.76)	1.24 (1.02; 2.55)
**SMI**	p = 0.192	p = 0.190
Adequate SMI (n = 69)	1	1
Low SMI^b^ (n = 15)	2.18 (0.68;7.05)	2.38 (0.65;8.75)
**SMD**	**p = 0.034**	**p = 0.015**
Adequate SMD (n = 70)	1	1
Low SMD^c^ (n = 14)	4.35 (1.12;16.97)	7.82 (1.5;40.88)
**Low Function + Muscle impairment**^d^	**p = 0.042**	**p = 0.022**
No (n = 68)	1	1
Yes (n = 16)	3.58 (1.05;12.23)	5.74 (1.28;25.64)

OR: Odds ratio; CI: confidence interval; BMI: body mass index; VAT: visceral adipose tissue SMI: skeletal muscle index; SMD: skeletal muscle radiodensity.

^a^Adjusted model: by age, gender and tumor stage;

^b^Low SMI: < 43 cm^2^/m^2^ (BMI < 25 kg/m^2^) or SMI < 53 cm^2^/m^2^ (BMI ≥ 25 kg/m^2^) for men; SMI < 41 cm^2^/m^2^ for women;

^c^Low SMD: SMD < 41 HU (BMI < 24.9 kg/m^2^) or < 33 HU (BMI ≥ 25 kg/m^2^);

^d^Low function + muscle impairment: low HGS + low SMI or low SMD.

When serious complications were considered (CDC ≥ III) (Table 5), obesity and SMD were predictive factors of postoperative complications, but after adjustments for confounding variables, only SMD remained as a predictive factor of risk.

**Table 5:** Logistic binary regression model analysis of factors associated with severe postoperative complications within 30 days after surgery in patients with gastric and colorectal cancer (n = 84).

	Complications Grade ≥ 3 (n = 14; 16.7%)	
Independent variables	Unadjusted	Adjusted^a^
	OR (95% CI)	OR (95% CI)
**BMI**	**p = 0.04**	p = 0.079
BMI < 30 kg/m^2^(n = 18)	1	1
BMI ≥30 kg/m^2^ (n = 66)	3.63 (1.06;12.37)	3.67 (0.86;15.65)
**Visceral obesity**	p = 0.095	p = 0.083
VAT ≤163.8/80.1 cm^2^ (n = 56)	1	1
VAT >163.8/80.1cm^2^ (n = 37)	1.18 (0.96;1.45)	1.14 (0.87; 1.33)
**SMI**	p = 0.703	p = 0.727
Adequate SMI (n = 69)	1	1
Low SMI^b^ (n = 15)	1.32 (0.32;5.45)	1.32 (0.28;6.29)
**SMD**	**p = 0.045**	**p = 0.046**
Adequate SMD (n = 70)	1	1
Low SMD^c^ (n = 14)	3.77 (1.03;13.79)	5.62 (1.03;30.54)
**Low Function + Muscle impairment**^d^	p = 0.326	p = 0.383
No (n = 68)	1	1
Yes (n = 16)	1.93 (0.52;7.21)	1.99 (0.42;9.37)

OR: Odds ratio; CI: confidence interval; BMI: body mass index; SMI: skeletal muscle index; SMD: skeletal muscle radiodensity.

^a^Adjusted model: by age, gender and tumor stage;

^b^Low SMI: < 43 cm^2^/m^2^ (BMI < 25 kg/m^2^) or SMI < 53 cm^2^/m^2^ (BMI ≥ 25 kg/m^2^) for men; SMI < 41 cm^2^/m^2^ for women;

^c^Low SMD: SMD < 41 HU (BMI < 24.9 kg/m^2^) or < 33 HU (BMI ≥ 25 kg/m^2^);

^d^Low function + muscle impairment: low HGS + low SMI or low SMD.

## Discussion

The preoperative nutritional assessment has gained importance in the treatment of patients undergoing surgery in order to identify risk factors that may be predictors of worse outcomes [15]. The main finding of this study was that low SMD was the best predictive factor associated with severe short-term postoperative complications in patients with gastric and colorectal cancer. For general surgical complications (CDC scale ≥ II), the presence of obesity, visceral obesity, and the presence of low function + muscle impairment also showed a significant association of risk.

Although there is no doubt that muscle mass depletion and malnutrition are considered surgical risks, methodological differences between studies can lead to confusion in analysis and result interpretation. The inclusion of patients with different surgical objectives (curative or palliative), type of surgery (laparoscopic or open procedure), and the number of surgical procedures in the same surgery, for example, can lead to inappropriate conclusions. Thus, in the present study, we included only patients that underwent curative surgery in an open procedure, and with that, we have obtained a more homogeneous sample.

Another factor that leads to confusion in the interpretation of the result between different studies is the fact that the presence of complications is determined by the CDC scale. While some studies consider surgical complications when CDC scale ≥ II [8, 21, 41, 42], others consider complications to be major only when CDC scale ≥ III [29, 43–45]. Therefore, in the present study, we analyzed the impact of the variables of nutritional status and body composition on the prediction of surgical risk using both classifications (general and major complications). In a retrospective study with 115 patients who underwent initial hepatectomy for colorectal liver metastasis, Horii et al. [43] described general (CDC scale ≥ II) and major (CDC scale ≥ III) complications in their patients, but the multivariate analysis was performed only with patients with major complications.

In the present study, patients classified as obese by BMI had a higher risk of general complications. In fact, more than half of the sample was overweight or obese, and it is interesting to remember that these characteristics are risk factors for the development of colorectal and gastric cancer [4]. Performing a surgical procedure in an obese patient requires more attention when manipulating a more voluminous mesentery with also increased difficulty in properly visualizing and identifying the surgical planes and blood vessels, and this peculiarity may explain the greater risk of complications for them [8]. However, the BMI value in the evaluation of hospitalized patients is questionable in the literature [46, 47], because they may have muscle mass deficit (sarcopenic obesity). In the present study, no patient was classified with this condition.

Our results showed that PG-SGA and cachexia were not associated with short-term surgical complications. Although about 38% of patients had cachexia, most of the patients were classified as well-nourished by PG-SGA. The good nutritional status found in the sample can also be attributed to the fact that the minority of the patients had previously undergone chemotherapy and radiotherapy, treatments with a negative impact on the nutritional status [46]. As the definition of cachexia allows different factors (weight loss, sarcopenia, and inflammation) to be present whether isolated or associated [35], and weight loss is an information that relies on memory, further studies are needed to investigate the effects of the different components on the surgical prognosis.

Although the risk associated with sarcopenia is not prohibitive for surgery, patients with low SMI/SMD require closer vigilance during their postoperative course. Several studies have evaluated body composition related to post-surgical complications in cancer patients. Recently, Xiao et al. showed that both low SMI and low SMD were associated with a higher risk of postsurgical complications, and short-term and long-term mortality in patients with colorectal cancer [48]. In a similar population of the same nationality, using the same cut-off points for low SMI, Maurício et al. [42] observed that low SMI and low muscle strength were associated with short-term postoperative complications in colorectal cancer patients. These results are different from those of the present study, probably because they have not included gastric cancer patients and the follow-up of these patients was curtailed by their hospital discharge, as well as in the majority of the studies [7, 26, 42, 49]. Jun Lu et al. [21] followed patients with gastric cancer after gastrectomy for a mean period of 64 months, and found that the SMI and SMD were associated with complications and a worse prognosis. However, the authors did not evaluate muscle strength. Margadant et al. [23], as in the present study, followed-up 373 older adult patients with colorectal cancer for 30 days after surgery, and observed that low SMD was associated with major postoperative complications.

Recently, Tankel et al. [50] published results of their retrospective single-center study with 580 surgical colorectal patients. Similar to the present study, considering major complications, low SMD was a predictor of complications, but not low SMI. SMD reflects the lipid content of the muscle, and SMI reflects the skeletal muscle volume or mass and it is measured as the skeletal muscle area divided by body height squared [51]. One possible explanation for this could be that the increase in lipid content of the muscle (myosteatosis) occurs before the decline in the size of muscle mass, and the deterioration of muscle is often occult, particularly in the presence of obesity [48]. Also, CT-based calculation allows for early detection of reduction in HU (SMD) while the muscle area remains unchanged; thus decrease in SMD is detected earlier than the corresponding decrease in SMI [52]. On the same way, Xiao et al. [24] observed that low SMD, but not low SMI, were associated with pre-existing comorbidities, suggesting a pioneer shared mechanism between them and fat infiltration into the muscle.

Regarding body adiposity, few studies have evaluated the effects of visceral fat on postoperative complications. Although the present study found that the patients who developed general complications had a higher amount of VAT compared to patients without them, this result was not observed for major complications. Chen et al. [8] evaluated the effects of visceral fat on post-surgical complications in patients with colorectal cancer, and they also observed statistically significant associations. They can be attributed to the fact that VAT secretes cytokines that systemically alter the immune, metabolic and endocrine system, influencing the body response to surgical stress, and its excess leads to an exacerbation of the post-surgical acute phase inflammatory response, affecting the immune system and resulting in worse outcomes [53].

Our study has some possible limitations. Although it is recognized that contrast infusion during CT analysis can increase muscle radiodensity measurements [54], we do not control this factor. However, because of the magnitudes of the differences are relatively small, the effect of the sweep phase on the accuracy of the results must be determined. The cut-off points adopted for classifying low SMI and low SMD were not proposed for the Brazilian population, and ethnic characteristics may influence this analysis. We recognize that the population in our study included only patients who performed open surgical procedure, not including laparoscopic surgery, and thus, future studies comparing these different surgical techniques are necessary. Finally, it is relevant to highlight that this is a single-center study with a small number of patients with heterogeneous cancer sites, so the results need to be analyzed with caution before being extrapolated to the general population.

In conclusion, the findings suggest that the presence of obesity, visceral obesity, low function + muscle impairment, and low SMD were associated and may predict an increased risk of short-term postoperative complications following gastric and colorectal cancer surgery, even in a sample with low nutritional risk. Low SMD also identifies individuals at risk of major complications. Thus, it is recommended to evaluate body composition by CT images, when available, before performing the surgical procedure, which may help to screen patients at higher risk of developing complications, thereby helping the multidisciplinary team to prepare the patient and reduce the risks arising from this therapeutic modality. The development of automated body composition analysis may be a reality in the next future, making possible the inclusion of body composition assessment from the CT-images as a routine in pre-operative care. For this, it is suggested that hospitals and the multidisciplinary teams invest in technology, training and time to analyze the images, aimed at improving patient care and supporting prior nutritional interventions to reduce surgical risk. Also, we suggest that future studies focus on comparing the role of muscle mass abnormalities on adverse outcomes between surgeries and less invasive procedures during cancer treatment.
